# Tooth loss is a complex measure of oral disease: Determinants and methodological considerations

**DOI:** 10.1111/cdoe.12391

**Published:** 2018-06-29

**Authors:** Simon Haworth, Dmitry Shungin, So Young Kwak, Hae‐Young Kim, Nicola X. West, Steven J. Thomas, Paul W. Franks, Nicholas J. Timpson, Min‐Jeong Shin, Ingegerd Johansson

**Affiliations:** ^1^ Medical Research Council Integrative Epidemiology Unit Department of Population Health Sciences Bristol Medical School University of Bristol Bristol UK; ^2^ Bristol Dental School University of Bristol Bristol UK; ^3^ Department of Odontology Umeå University Umeå Sweden; ^4^ Department of Public Health Sciences BK21PLUS Program in Embodiment: Health‐Society Interaction Graduate School Korea University Seoul Republic of Korea; ^5^ Department of Clinical Sciences, Genetic and Molecular Epidemiology Unit Lund University Skåne University Hospital Malmö Malmö Sweden; ^6^ Department of Public Health and Clinical Medicine Umeå University Umeå Sweden; ^7^ Department of Nutrition Harvard T. H. Chan School of Public Health Boston MA USA

**Keywords:** caries, epidemiology, periodontal diseases, tooth loss

## Abstract

**Objectives:**

Counts of missing teeth or measures of incident tooth loss are gaining attention as a simple way to measure dental status in large population studies. We explore the meaning of these metrics and how missing teeth might influence other measures of dental status.

**Methods:**

An observational study was performed in 2 contrasting adult populations. In total, 62 522 adult participants were available with clinically assessed caries and periodontal indices from the Swedish arm of the Gene‐Lifestyle Interactions and Dental Endpoints Study (GLIDE) and the Korea National Health and Nutrition Examination Survey (KNHANES) in the Republic of Korea. Longitudinal measures of tooth loss were available for 28 244 participants in GLIDE with median follow‐up of 10.6 years.

**Results:**

In longitudinal analysis, hazard for tooth loss was associated with baseline dental status (previous tooth loss, periodontal status and caries status) and socio‐demographic variables (age, smoking status and highest educational level). Analysis of cross‐sectional data suggested that indices of caries exposure were not independent of periodontal status. The strength and direction of association varied between groups, even for measures specifically intended to avoid measuring tooth loss. Individuals with impaired periodontal health (community periodontal index [CPI] 3 or higher in any sextant) had higher standardized decayed and filled surfaces (DFS; number of DFS divided by total number of tooth surfaces) in GLIDE (incidence risk ratio [IRR] 1.05 [95% CI: 1.04, 1.07], but lower standardized DFS in KNHANES (IRR: 0.95 [0.92, 0.98]) than individuals with better periodontal health (CPI <3 in all sextants).

**Conclusions:**

Incident tooth loss is a complex measure of dental disease, with multiple determinants. The relative importance of dental caries and periodontal disease as drivers of tooth loss differs between age groups. Measures of dental caries exposure are associated with periodontal status in the studied populations, and these associations can be population‐specific. Consideration of the study‐specific properties of these metrics may be required for valid inference in large population studies.

## 1 INTRODUCTION

Associations between oral health and health status are of interest to the public, clinicians and the research community. Epidemiological studies investigating these relationships require measures of oral disease, which ideally would be comprehensive, specific and validated. Nevertheless, the practicalities of dental examinations in large‐scale studies mean that simpler measures of dental status may be used. Several studies have reported that having fewer teeth at baseline or losing teeth during study follow‐up increases the risk of a range of adverse health outcomes.^1^, ^2^, ^3^, ^4^, ^5^, ^6^, ^7^ While there is growing interest in tooth loss as an epidemiological measure of dental status, valid inference from these studies requires an understanding of what is represented by tooth loss.

Observational studies report dental caries and periodontal disease are the most common indications for the extraction of permanent teeth, but the relative importance may change between studies with evidence for effects of age (the reasons for tooth loss differ between age groups), period (the reasons for tooth loss change with time), and population (the reasons for tooth loss differ by socioeconomic‐status and culture).^8^, ^9^, ^10^, ^11^, ^12^, ^13^ Thus, studies examining tooth loss would ideally be designed to allow the relative importance of dental caries and periodontal disease to vary between populations and older and younger participants in the same population.

To clarify the meaning of tooth loss in the epidemiologic context, it would be helpful to use longitudinal data. A few studies report on tooth survival over time or trends in repeated cross‐sectional samples^14^, ^15^, ^16^, ^17^, ^18^, ^19^ but most rely on self‐reported tooth loss with limitations in validity,^14^, ^16^, ^17^ or are performed in selected patient groups with limitations in generalizability into the general population.^19^, ^20^ To date, the largest published longitudinal analysis of changes in dental status comes from the Dunedin study, a prospective cohort study of approximately 1000 participants born in 1972/1973.^21^, ^22^, ^23^ This study provides insight up to age 38, where levels of tooth loss remain low.^21^ Thus, large‐scale studies with population‐based, clinically assessed, longitudinal data in participants older than 40 years where tooth loss may occur,^24^, ^25^ are needed but to date such studies are scarce.^26^

The reasons for loss of permanent teeth (caries, periodontal disease or other causes, such as trauma or orthodontic treatment) would ideally be obtained at the time of the loss, but information may be inaccessible. Thus, missing teeth may bias inferences of both caries and periodontal disease. For periodontal disease, susceptible teeth might be lost at the time of examination, and for caries, missing teeth/surfaces are usually included in the scores leading to potential under‐ and overestimation, respectively.^27^, ^28^ In addition to biased single‐disease estimates, indices of caries and periodontal disease exposure may become correlated as teeth are lost. Teeth lost through periodontal disease affect the numerator or denominator of WHO caries indices, and teeth lost due to caries may have been affected by unrecognized periodontal symptoms too. The extent to which measures of periodontal status and caries experience are correlated requires characterization of the magnitude and direction in different populations and age groups.

In summary, tooth loss is entering routine use as an epidemiological measure of dental health, but the properties of this measure are not well understood. We aimed to examine the drivers of tooth loss and analytical implications of using different dental indices by: (i) describing the major determinants of incident tooth loss from over 290 000 person‐years of participant‐level dental follow‐up; and (ii) identifying whether caries experience (as represented by WHO caries indices) is independent of periodontal status in 2 large and adult populations with contrasting diet, healthcare systems and ethnicity.

## 2 METHODS

This study was conducted in 2 independent populations, in Sweden and the Republic of Korea (hereafter “Korea”). The Swedish arm included participants from the Gene‐Lifestyle Interactions and Dental Endpoints (GLIDE) study which previously contributed to the GLIDE consortium,^29^ while the Korean arm included participants from the Korea National Health and Nutrition Examination Survey (KNHANES).

Gene‐Lifestyle Interactions and Dental Endpoints dental data were obtained from clinical examinations performed by dentists working in Public Dental Service clinics in the County of Västerbotten, Northern Sweden and recorded in electronic dental records. Data from surface‐level assessment of caries, restorations, missing teeth and periodontal examination are stored in a regional register. The participants were recruited from the population‐based Västerbotten Intervention Program (VIP)^30^ by linkage to the dental register using a 12‐digit personal identity number. Those with a dental record available within a 5‐year window from a VIP visit were included. Participants aged ≥18 years were included with no exclusions for maximum age. For longitudinal analysis, participants needed to have multiple outcome data available. Information on demographic characteristics and smoking behaviour was from the VIP records. Detailed information is given in the Appendix S1.

The KNHANES is a national cross‐sectional survey which includes a new sample of approximately 10 000 individuals each year. KNHANES aims to achieve a sample which is representative of the noninstitutionalized civilian population of Korea, using a stratified, multistage probability cluster study design. KNHANES performs health interviews and health examinations in a mobile examination centre with a nutritional survey 1 week later in participants' homes. Between 2008 and 2014, the health examination included clinical assessment of dental caries experience with surface‐level dental charting for all adult participants. Periodontal examination was undertaken during 2008‐2010 and 2012‐2014 using index teeth,^31^ as described in the Appendix S1. The KNHANES study and protocol is described in full elsewhere.^32^ Participants were eligible if they were aged 18 years or older at the time of screening and had exposure, outcome and covariate data available, with no exclusions for maximum age.

The Community Periodontal Index (CPI) is a 5 level measure of periodontal health which is used to screen for periodontal treatment need and scored separately for 6 regions (sextants) of the mouth.^33^ Higher scores indicate worse periodontal health. Participants with a CPI score of 3 or 4 in one or more sextants were classified as having impaired periodontal health. In GLIDE some participants had full mouth pocket charting data rather than CPI scores. These participants were classified as having impaired periodontal health if 4 teeth in the mouth had pocketing of at least 4 mm, or if any tooth in the mouth had pocketing of at least 5 mm. Indices for number of teeth, number of tooth surfaces, decayed missing and filled surfaces (DMFS), decayed missing and filled teeth (DMFT), decayed and filled surfaces (DFS) and standardized DFS (standardized DFS) were derived from surface‐level electronic dental charting excluding third molar teeth.

The demographic variables used in the analyses were age, sex and highest educational level (as a proxy for socioeconomic status). Smoking behaviour was also included using self‐reported questionnaire data.

Longitudinal analysis was performed using a Weibull survival model using the “streg, dist(weibull)” function in the statistical package Stata. The exposure variables were baseline demographic and dental variables (including number of teeth) and the outcome was prospective tooth loss. All participants were considered to have entered the study at their first dental examination. At each subsequent dental examination, the number of teeth was compared to the number of teeth at baseline, and participants who lost 1 or more teeth were classified as having undergone failure. The failure time was entered as the time elapsed (in years) between entering the study and the first tooth loss event. Individuals who had not lost a tooth by the last available dental examination were censored, and time was entered as the time elapsed (in years) between entering the study and the final dental examination. All models were fitted with 3 age strata (<45 years, ≥45 to <55 years, ≥55 years at baseline) to allow influence of predictors to vary across strata of age. Univariate models were used to obtain unadjusted hazard ratios for potential predictors, before multiple predictors were fitted simultaneously in a multivariate model.

Cross‐sectional analysis tested the association between periodontal status and WHO caries indices. Here periodontal status was considered the exposure and multiple outcomes (eg DMFS, DFS) were tested. All WHO caries indices represent count data and were therefore analysed using Poisson regression, implemented using the “poisson” function in the statistical package Stata. The beta coefficients from this analysis are difficult to interpret without transformation and were therefore exponentiated. Following standard terminology, we describe the transformed effect estimates as incidence risk ratios (IRR), however, in the context of this cross‐sectional analysis, IRR represents the fully adjusted ratio of a given caries index in exposed individuals compared to nonexposed individuals. For example, an incidence risk ratio of 1.5 for DMFS means the expected DMFS index count in participants with CPI ≥3 is 1.5 times the expected DMFS index count in participants with CPI <3.

## 3 RESULTS

The GLIDE and KNHANES populations included in cross‐sectional analysis had comparable sample sizes, mean age and proportion with university‐level education (Table 1). Both populations had greater representation from female than male participants. The KNHANES population contained a greater proportion of active smokers but had lower levels of oral disease (DMFS, proportion with CPI ≥3 and missing teeth) than the GLIDE population. In longitudinal analysis in GLIDE around one‐third of participants experienced tooth loss during follow‐up, with median time at risk of approximately 12 years.

**Table 1:** Demographic characteristics of the final samples included in the analysis

	GLIDE Sweden		KNHANES
	Cross‐sectional	Longitudinal	
Included participants with baseline observations (N)	28 691	28 244	33 831
Follow‐up observations (N)		232 905	
Total observations(N)^a^		261 149	
Time at risk (y)			
Median per‐participant		11.7	
Total		298 120	
Sex, % male	49.1	49.2	49.4
Age, years at study baseline (mean [SD])	49.7 (8.6)	45.0 (8.9)	49.2 (16.5)
Proportions in age groups			
Under 45 y	35.1	35.1	42.8
45‐54.9 y	34.1	34.1	19.2
55 y and older	30.8	30.8	38.0
Dental status at baseline			
Missing teeth (mean [SD])	2.0 (3.2)	1.8 (3.0)	0.71 (6.0)
DFS (mean [SD])	33.3 (19.3)	31.4 (19.2)	11.8 (12.0)
DMFS (mean [SD])	43.2 (25.9)	40.1 (25.2)	22.5 (22.7)
CPI ≥3 (%)	52.0	51.8	27.7
Smoking status			
Current smoker	12.3	12.2	20.5
Not current smoker	87.7	87.8	79.5
Education, % University level	26.9	26.9	29.6

CPI, community periodontal index; DFS, decayed and filled surfaces; GLIDE, Gene‐Lifestyle Interactions and Dental Endpoints Study; KNHANES, Korea National Health and Nutrition Examination Survey.

a Total observations is the sum of baseline and follow‐up observations included in longitudinal modelling.

### 3.1 GLIDE longitudinal analysis—dental status as a predictor of tooth loss

Participants with CPI 3 or higher had greater hazard for tooth loss than participants with CPI <3 and this effect was larger in younger participants than older participants (Table 2). Each additional decayed or filled tooth surface at study baseline was associated with increasing hazard for tooth loss in individuals aged under 45 years but was modelled to have minimal effect on hazard in individuals aged 55 years or older. Hazard for tooth loss was higher in individuals who had previously lost teeth than individuals who had not previously lost any teeth despite adjustment for periodontal status and baseline DFS, with the largest effect in individuals under 45 years who had already lost 7 or more teeth.

**Table 2:** Unadjusted and fully adjusted hazard ratios for dental status as a predictor of tooth loss

Exposure	Age group	Reference (for categorical exposures)	Unadjusted hazard ratio (95% CI)	Unadjusted *P* value	Fully adjusted hazard ratio (95% CI)	Fully adjusted *P* value
CPI3 or higher	Under 45	CPI <3	1.64 (1.50, 1.79)	<.0001	1.41 (1.28, 1.55)	<.0001
	45‐54.9		1.51 (1.40, 1.69)	<.0001	1.28 (1.18, 1.38)	<.0001
	55 or older		1.38 (1.28, 1.48)	<.0001	1.22 (1.14, 1.32)	<.0001
	Combined		1.48 (1.42, 1.55)	<.0001	1.29 (1.24, 1.36)	<.0001
Baseline DFS	Under 45	‐	1.02 (1.02, 1.03)	<.0001	1.01 (1.01, 1.02)	<.0001
	45‐54.9		1.01 (1.01, 1.01)	<.0001	1.01 (1.00, 1.01)	<.0001
	55 or older		1.00 (1.00, 1.00)	.004	1.00 (1.00, 1.00)	.013
	Combined		1.01 (1.01, 1.01)	<.0001	1.00 (1.00, 1.01)	<.0001
1‐2 missing teeth at baseline	Under 45	No missing teeth at baseline	1.59 (1.43, 1.78)	<.0001	1.38 (1.24, 1.54)	<.0001
	45‐54.9		1.39 (1.28, 1.51)	<.0001	1.18 (1.08, 1.28)	<.0001
	55 or older		1.15 (1.05, 1.26)	<.0001	1.04 (0.95, 1.14)	.411
	Combined		1.34 (1.27, 1.41)	<.0001	1.17 (1.10, 1.23)	<.0001
3‐6 missing teeth at baseline	Under 45		1.25 (1.09, 1.44)	<.0001	1.17 (1.02, 1.35)	.026
	45‐54.9		1.55 (1.41, 1.70)	<.0001	1.21 (1.10, 1.33)	<.0001
	55 or older		1.46 (1.34, 1.60)	<.0001	1.16 (1.06, 1.27)	.001
	Combined		1.52 (1.44, 1.61)	<.0001	1.22 (1.15, 1.29)	<.0001
7 or more missing teeth at baseline	Under 45		5.14 (3.27, 8.09)	<.0001	2.50 (1.57, 3.99)	<.0001
	45‐54.9		3.87 (3.25, 4.62)	<.0001	2.12 (1.75, 2.56)	<.0001
	55 or older		1.85 (1.68, 2.03)	<.0001	1.22 (1.10, 1.36)	<.0001
	Combined		2.18 (2.02, 2.35)	<.0001	1.43 (1.32, 1.56)	<.0001

The multivariable model includes adjustment for age and sociodemographic parameters given in Table 3.

CPI, community periodontal index; DFS, decayed and filled surfaces.

### 3.2 GLIDE longitudinal analysis—age and sociodemographic predictors of tooth loss

Each 1‐year increase in baseline age was associated with increasing hazard for tooth loss (Table 3). Participants aged 55 years and older had a greater increment in hazard for each 1‐year increase in baseline age than younger participants. Current smokers had greater hazard for tooth loss than nonsmokers. Higher educational attainment was associated with decreased hazard for tooth loss.

**Table 3:** Unadjusted and fully adjusted hazard ratios for age and sociodemographic variables as a predictor of tooth loss

Exposure	Age group	Reference (for categorical exposures)	Unadjusted hazard ratio (95% CI)	Unadjusted *P* value	Fully adjusted hazard ratio (95% CI)	Fully adjusted *P* value
Age 45‐54.9 y	‐	Under 45	1.37 (1.29, 1.45)	<.0001	1.79 (1.71, 1.81)	<.0001
Age ≥55 y			2.13 (2.01, 2.24)	<.0001	1.48 (1.42, 1.55)	<.0001
Baseline age (1 y increment within age group)	Under 45	‐	1.08 (1.07, 1.10)	<.0001	1.04 (1.03, 1.06)	<.0001
	45‐54.9		1.10 (1.09, 1.11)	<.0001	1.07 (1.06, 1.08)	<.0001
	55 or older		1.09 (1.08, 1.10)	<.0001	1.08 (1.07, 1.09)	<.0001
	Combined		1.09 (1.08, 1.10)	<.0001	1.07 (1.07, 1.08)	<.0001
Current smoker	Under 45	Nonsmoker	2.53 (2.22, 2.88)	<.0001	1.80 (1.57, 2.07)	<.0001
	45‐54.9		2.08 (1.89, 2.28)	<.0001	1.57 (1.43, 1.74)	<.0001
	55 or older		1.74 (1.60, 1.89)	<.0001	1.56 (1.43, 1.70)	<.0001
	Combined		1.87 (1.98, 2.10)	<.0001	1.62 (1.53, 1.71)	<.0001
Groundschool or equivalent	Under 45	Elementary school	0.65 (0.51, 0.84)	.001	0.79 (0.62, 1.02)	.067
	45‐54.9		0.85 (0.75, 0.95)	.006	0.82 (0.73, 0.92)	.001
	55 or older		0.87 (0.80, 0.93)	<.0001	1.01 (0.94, 1.09)	.754
	Combined		0.84 (0.79, 0.90)	<.0001	0.92 (0.87, 0.98)	.011
Highschool or equivalent	Under 45	Elementary school	0.55 (0.47, 0.64)	<.0001	0.76 (0.65, 0.90)	.001
	45‐54.9		0.65 (0.59, 0.72)	<.0001	0.91 (0.82, 1.01)	.072
	55 or older		0.83 (0.76, 0.91)	<.0001	1.10 (1.01, 1.21)	.033
	Combined		0.83 (0.73, 0.91)	<.0001	0.98 (0.92, 1.04)	.467
University/College or equivalent	Under 45	Elementary school	0.46 (0.39, 0.55)	<.0001	0.70 (0.58, 0.84)	<.0001
	45‐54.9		0.75 (0.69, 0.82)	<.0001	0.85 (0.76, 0.96)	.01
	55 or older		0.61 (0.54, 0.68)	<.0001	0.99 (0.90, 1.09)	.856
	Combined		0.66 (0.62, 0.70)	<.0001	0.91 (0.85, 0.97)	.006
Female sex	Under 45	Male sex	1.09 (1.00, 1.20)	.055	1.11 (1.01, 1.22)	.032
	45‐54.9		1.16 (1.08, 1.25)	<.0001	1.11 (1.03, 1.19)	.005
	55 or older		1.00 (0.94, 1.06)	.962	0.97 (0.91, 1.03)	.355
	Combined		1.07 (1.03, 1.12)	.001	1.04 (1.00, 1.08)	.071

The fully adjusted estimates are obtained from a multivariable model which also includes adjustment for parameters given in Table 2.

### 3.3 Analysis of cross‐sectional data

We examined the associations between periodontal status and WHO caries indices in GLIDE and KNHANES to evaluate whether these relationships are stable or population‐specific. Multiple caries indices were associated with periodontal status (Table 4). In GLIDE, participants with CPI 3 or higher had greater incidence risk for caries traits than participants with better periodontal health. These associations were consistent in direction across age groups but were more pronounced in younger participants. KNHANES participants with CPI 3 or higher had similar incidence risk for DMFS as participants with better periodontal health, but the combined estimate masked potential age‐specific effects. Younger participants (aged under 45 years) with CPI 3 or higher had higher incidence risk for DMFS than participants with better periodontal health while older participants (≥55 years) with CPI 3 or higher had reduced incidence risk than participants with better periodontal health. Overall, KNHANES participants with CPI 3 or higher had reduced incidence risk for DFS and standardized DFS than participants with better periodontal health (Table 4).

**Table 4:** Differences in caries indices between periodontal cases and controls

Age group	% of cases	Number	Fully adjusted ratio of index in cases: controls (95% CI)					
			DMFS	DMFT	DFS	Standardized DFS	Number of surfaces	Number of teeth
GLIDE Cross‐sectional analysis—Cases defined as CPI ≥3 in one or more sextants or 4 mm pocketing around 4 or more teeth or 5 mm pocketing around 1 or more teeth								
Under 45	36.4	10 073	1.09 (1.06, 1.12)	1.05 (1.03, 1.07)	1.07 (1.04, 1.10)	1.08 (1.05, 1.11)	0.99 (0.99, 1.00)	0.99 (0.99, 1.00)
45‐54.9	53.1	9794	1.07 (1.04, 1.09)	1.02 (1.01, 1.04)	1.03 (1.01, 1.05)	1.04 (1.02, 1.07)	0.99 (0.98, 0.99)	0.99 (0.98, 0.99)
55 or older	68.6	8824	1.03 (1.01, 1.05)	1.02 (1.01, 1.03)	1.05 (1.03, 1.07)	1.04 (1.02, 1.06)	1.00 (0.99, 1.01)	1.00 (1.00, 1.01)
Overall	52.0	28 691	1.06 (1.05, 1.07)	1.03 (1.02, 1.04)	1.05 (1.04, 1.06)	1.05 (1.04, 1.07)	0.99 (0.99, 1.00)	0.99 (0.991, 1.00)
KNHANES cross‐sectional analysis—Cases defined as CPI ≥3 in one or more sextants								
Under 45	14.0	14 486	1.11 (1.06, 1.17)	1.01 (0.97, 1.05)	0.96 (0.91, 1.02)	0.98 (0.92, 1.03)	0.98 (0.98, 0.99)	0.99 (0.98, 0.99)
45‐54.9	38.8	6500	1.15 (1.08, 1.21)	1.08 (1.03, 1.13)	0.96 (0.90, 1.02)	0.97 (0.91, 1.04)	0.97 (0.97, 0.98)	0.97 (0.97, 0.98)
55 or older	46.2	12 845	0.94 (0.91, 0.96)	0.94 (0.92, 0.96)	1.02 (0.97, 1.07)	0.98 (0.93, 1.03)	1.04 (1.02, 1.05)	1.03 (1.02, 1.05)
Overall	27.7	33 831	0.99 (0.97, 1.01)	0.95 (0.93, 0.97)	0.97 (0.94, 1.00)	0.95 (0.92, 0.98)	1.01 (1.01, 1.02)	1.01 (1.01, 1.02)

Differences are obtained from a multivariable Poisson regression model incorporating adjustment for age, sex, smoking status and highest educational level (as a proxy for socioeconomic status). Analysis in KNHANES also incorporated adjustment for year of participation in KNHANES.

CPI, community periodontal index; DFS, decayed and filled surfaces; DMFT, decayed missing and filled teeth; DMFS, decayed missing and filled surfaces; GLIDE, Gene‐Lifestyle Interactions and Dental Endpoints Study; KNHANES, Korea National Health and Nutrition Examination Survey.

## 4 DISCUSSION

Counts of missing teeth or incident tooth loss are gaining traction as a low‐cost and simple way to measure dental status at scale. We evaluated incident tooth loss and number of teeth in relation to potential causes and confounders and identified that tooth loss is a complex measure of oral health, requiring caution in both biological interpretation as well as extrapolation between different age groups or populations.

The main strengths of the study are the large sample size with clinically assessed data, representative populations and inclusion of longitudinal data. The Swedish GLIDE participants were recruited through a population‐based health screening programme (VIP) with a participation rate >65% and little evidence for selection bias.^34^ The KNHANES study is designed to be a representative sample of the noninstitutionalized Korean population and reports participation rates >70%. The study has limitations which should be considered. While there is value in considering 2 very different populations, this will increase heterogeneity in the estimates. Further, the index‐linked data in Sweden were from many dentists, rather than a calibrated team, and although most had their basic training in Sweden and all participated in mandatory supplementary training, both under‐ and over‐scoring would be present. Finally, teeth missing due to dental disease could not be distinguished from other causes, and CPI scores were used as a common basis for comparison across the 2 populations but these scores have limitations and do not provide a clinical diagnosis of periodontal disease.

We evaluated dental status as a predictor of tooth loss in a longitudinal analysis as well as the association between clinically assessed measures of dental status and tooth loss in 2 culturally distinct cohorts of middle aged and older men and women, which has not been performed before at large‐scale. Several findings agreed with other studies using self‐reported data or repeated cross‐sectional population samples, namely that the prevalence of tooth loss, impaired periodontal health and caries experience increase with age and that tooth loss was associated with age, smoking and educational level. The relationship between age and tooth loss appears complex and nonlinear. Each additional year of age at entering the GLIDE study was associated with a greater increment in hazard for older than younger participants. This is a concern for studies exploring the relationship between tooth loss and general health status, as many health outcomes have a nonlinear relationship with age too. Linear adjustment for age may be insufficient to prevent spurious association between tooth loss and health outcomes.

Worse periodontal health (CPI ≥3) was associated with higher standardized DFS in GLIDE but lower standardized DFS in KNHANES, which may reflect population‐specific bias from tooth loss. In GLIDE (where periodontal disease is common and associated with tooth loss) periodontal status may increase the denominator and inflate estimates of caries in people with worse periodontal status. By contrast, in KNHANES (where levels of tooth loss and periodontal disease are lower) the main determinant of missing teeth is likely to be dental caries and “accounting” for periodontal tooth loss leads to a bias in the opposite direction. This variability may be influenced by genetic, lifestyle and health care structures altering the balance between competing and interacting risk factors for tooth loss.^34^, ^35^, ^36^, ^37^ To catalogue the factors mediating between‐population variability in the relationships described here will require a greater number of contrasting populations with measurement of a range of features pertinent to oral health and falls outwith the scope of the present study.

The number of missing teeth at study baseline was associated with subsequent tooth loss, even in a model which incorporated adjustment for baseline CPI and DFS. Previous tooth loss may capture complex causes of tooth loss which act independently of dental disease. For example, previous tooth loss may indicate that a participant or the dentist prefer extraction over other treatment options for a particular clinical scenario, and therefore predicts extraction as the preferred treatment choice in the future. Alternatively, this result may demonstrate that DFS and CPI are imperfect measures which do not capture the full biological spectrum of dental caries or periodontal disease. The latter argument appears plausible considering the recognized limitations of CPI and DFS, which have been discussed previously in the literature.^28^, ^38^

Indices of dental status should provide precise and unbiased estimates for the dental diseases and accounting for bias introduced by missing teeth may refine the existing systems. More than 10 years ago a modification was proposed to the DMFS index which would take into account information from the previous or typical status of other teeth to decide how many surfaces of a missing tooth were likely to be carious.^28^ It may be timely to revisit this approach and think about ways to integrate knowledge of caries status, periodontal status and the normative relationships between these traits to define a reasonable estimate for what missing teeth represent in the individual. To estimate the burden of periodontal disease at a group or population level some consideration of the level of missing teeth would be informative, especially in middle aged and older people. Again, including reasonable prior knowledge, such as allocating a proportion of missing teeth as missing due to periodontal disease in patients where the empirical evidence points to a history of periodontal tooth loss may help. Retrospectively diagnosing previous dental disease which leads to tooth loss will be a challenge in population‐level studies but one deserving of the same status as diagnosing periodontal disease and caries present at the time of examination. We also suggest that the existence of age‐ and population‐specific properties of dental indices is an important factor to consider when drawing conclusions from epidemiological studies.

In conclusion, incident tooth loss is a complex measure of dental disease, with multiple determinants. The relative importance of dental caries and periodontal disease as drivers of tooth loss differs between age groups. Measures of dental caries exposure are associated with periodontal status in the studied populations, and these associations can be population‐specific. Consideration of the study‐specific properties of these metrics may be required for valid inference in large population studies.

## ETHICAL APPROVAL

This study was conducted in accordance with principles described in the Helsinki declaration and all additional requirements within the United Kingdom, Sweden and Korea. Data compilation for the GLIDE consortium was approved by the Regional Ethical Review Board (EPN) at Umeå University, Sweden (Dnr 2010‐387‐31M and 2011‐74‐32M. All participants have given written informed consent for use of data in research given that results are presented in an untraceable way. The KNHANES was approved by the Institutional Review Board (IRB) of the Korean Centers for Disease Control and Prevention (IRB: 2008‐04EXP‐01C, 20089‐01CON‐03‐2C, 2010‐02CON‐21C, 2012‐01EXP‐01‐2C, 2013‐07CON‐03‐4C, 2013‐12EXP‐03‐5C). All study participants provided informed written consent.

## CONFLICTS OF INTEREST

There are no conflicts of interest to declare.

## AUTHOR CONTRIBUTIONS

Simon Haworth contributed to conception, design, data analysis and interpretation, drafted and revised the manuscript; Dmitry Shungin contributed to conception, design, data acquisition, interpretation and critically revised the manuscript; So Young Kwak contributed to data acquisition, data analysis and interpretation; Hae‐Young Kim, contributed to data acquisition, data analysis and interpretation; Nicola West contributed to interpretation, critically revised the manuscript; Steven Thomas contributed to interpretation and critically revised the manuscript; Paul W Franks contributed to conception, design, data acquisition, interpretation and critically revised the manuscript; Nicholas Timpson contributed to conception, design, data analysis, interpretation and critically revised the manuscript; Min‐Jeong Shin contributed to design, data acquisition, interpretation and critically revised the manuscript; Ingegerd Johansson contributed to conception, design, data acquisition, data analysis, interpretation and critically revised the manuscript. All authors gave final approval.
