# Supplementary material for: Tooth loss is a complex measure of oral disease: Determinants and methodological considerations
Source: Community Dent Oral Epidemiol. 2018 Jun 29;46(6):555–62. doi: 10.1111/cdoe.12391 (PMC6282797; doi:10.1111/cdoe.12391)
Supplement: Supplementary file 1 [file CDOE-46-555-s001.docx]

**Appendix to ‘Tooth loss is a complex measure of oral disease: determinants and methodological considerations’**

**Authors:** Simon Haworth, Dmitry Shungin, So Young Kwak, Hae-Young Kim, Nicola X West, Steven J Thomas, Paul W Franks, Nicholas J Timpson, Min-Jeong Shin, Ingegerd Johansson.

**The Swedish Gene-Lifestyle Interactions and Dental Endpoints (GLIDE) database.**

The Swedish GLIDE database contains clinically assessed dental data and was established to facilitate research into dental caries and periodontal disease.

*Collection of dental data*

For the present study the eligible population in Swedish GLIDE were participants who took part in the Västerbotten Intervention Program (VIP),^1^ a population based health screening and intervention study in Northern Sweden. Dental data have been obtained from electronic dental records stored in registers at the Public Dental Service (Folktandvården) in the county of Västerbotten, Sweden. The electronic dental record is a full substitute for a paper dental record. Information is logged and updated each time a dentist performs a diagnostic procedure or treatment and is stored in real-time on servers located at the Public Dental Service headquarter. VIP participants who attended a dentist at a Public Dental Clinic in the region for a full mouth examination with surface-level documentation at least once from 1^st^ January 2000 to 16^th^ November 2015 were included. Linkage of the dental data with information from VIP with participation information was performed using a unique 12-digit Swedish personal identification number.

*Assessment of caries status and missing teeth*

Within each dental clinic, tooth level charting was undertaken using good light, mirrors, probes and radiographs where indicated. For present teeth, the charting records the tooth/restorative status of each surface, including caries, restorations and sound surfaces. For surfaces charted as showing evidence of caries, there is further information recorded on the extent of caries, classified as D1, D2 and D3 diagnostic thresholds. For restorations information is available on dental fillings, inlays and onlays. Fissure sealants are not recorded as fillings. For teeth charted as missing, data are not available on the reasons for tooth loss but the presence of replacements such as dental implants or bridges.

*Assessment of periodontal status*

Most participants in Swedish GLIDE have Community Periodontal Index (CPI) measures obtained as part of routine screening for periodontal disease. Some participants have full mouth pocket chart data instead of CPI scores, and some participants have both measures available.

*Derivation of per-mouth summary data for use in the present analysis.*

For the present study, caries experience was summarized using World Health Organization caries indices, which were derived centrally from surface-level data. First, third molar teeth were excluded, leaving 28 teeth. For the purposes of these summary indices, surfaces with initial carious lesions were not classified as decayed, whilst lesions requiring operative intervention (D2 and higher) were recoded as decayed. Following WHO guidelines, all missing teeth were included in deriving the DMFS and DMFT indices regardless of cause.^2^ Missing teeth or teeth with a crown restauration were calculated as 4 of 5 caries affected surfaces for premolars, and molars, respectively.

Periodontal status was summarized by two categories, representing better periodontal health or worse periodontal health. Any participant with CPI 3 or higher in any sextant was considered to have impaired periodontal health, while participants with CPI 2 or lower in all sextants were considered to have better periodontal health.

To include participants with full mouth pocket chart data in these definitions, pocket depth measures comparable to CPI scores were created and validated in participants with both CPI and full mouth pocket chart data available. CPI 3 or higher in any sextant was comparable to four teeth in the mouth with 4 mm pocketing or greater or one or more teeth in the mouth with 5 mm pocketing or greater.

Selection of dental data for inclusion in present analysis

This study included GLIDE participants who were originally recruited through the Västerbotten Intervention Programme (VIP) in Västerbotten county, northern Sweden. These individuals needed to have complete and valid data on caries and restorative status, periodontal status and relevant demographic and covariables as described in the main manuscript. Many participants in Swedish GLIDE have dental data from many clinical examinations spread over several years, whilst some participants in VIP have participated in health screening on more than one occasion and therefore have data available from multiple dates. Caries data from dental charting and periodontal data from CPI or pocket charting may be recorded on different dates. To identify the maximum sample size meeting the inclusion criteria for cross-sectional analysis, a sequential data merge process was undertaken.

**Dental data in in the KNHANES study.**

In KNHANES dental data were obtained by clinical examination as part of the study protocol and therefore includes individuals who do not routinely access dental care. Participants were asked whether they had experienced dental care during the previous year. The proportion who had accessed dental treatment were 60.3%, 30.7%, 63.3% and 39.9% in 2010, 2011, 2012 and 2013, respectively.^3^ Dental examination was performed in the mobile examination centre, which was staffed by a team of 4 public health dentists responsible for oral health examination. These dentists are provided by a combination of the Centers for Disease Control and 16 cities and provinces. Site conduct quality control is undertaken by the Korean Academy of Preventive Dentistry and Oral Health, who are responsible for surveyor training, site inspection and conformity assessment.

*Assessment of dental status.*

Surface-level dental charting was obtained. Each surface was classified as sound, active caries, filled missing (due to caries), missing (for any other reason), sealed, treated (due to any other reasons) or unerupted. In addition, prothesis such as bridges, partial dentures, complete dentures and implants were recorded.

*Assessment of periodontal status*

CPI scores were obtained from each sextant which had two or more natural teeth. If they were present, scores were obtained from index teeth in each sextant (17/16, 11, 26/27, 36/37, 31, 46/47). If index teeth were lost then adjacent teeth were examined instead.^4^

*Derivation of per-mouth summary data for use in the present analysis.*

In line with Swedish GLIDE, WHO caries indices were derived centrally from surface-level charting. Sealed surfaces were not considered carious. The same binary classification for periodontal status were used.

**Sensitivity analysis using an alternative definition of periodontal status.**

*Rationale*

In the main analysis CPI ≥3 was used to define individuals with impaired periodontal health. This definition could include participants with false pocketing related to gingivitis rather than periodontal attachment loss. To explore the possible impact of mis-classification, sensitivity analysis was undertaken using a more stringent definition of impaired periodontal health.

*Periodontal status definition in sensitivity analysis*

In KNHANES and GLIDE participants with CPI scores, participants with CPI 4 in one or more sextants to have impaired periodontal health, while participants with CPI 3 or lower in all sextants were considered to have better periodontal health. In GLIDE participants with full-mouth pocket charting, participants with one or more teeth in the mouth with 6 mm pocketing were considered to have impaired periodontal health. In Swedish GLIDE 26.3% of participants included in cross-sectional analysis and 26.0% of participants included in longitudinal analysis had CPI4 or equivalent pocket depth. In KNHANES, 6.6% of participants had CPI4.

*Results of sensitivity analysis.*

In longitudinal analysis, participants with CPI4 had greater hazard for tooth loss than remaining participants, with the largest effect sizes in younger participants (>45 years) (Table A1). This finding is consistent with the results of the main analysis, showing that impaired periodontal health is associated with incident tooth loss.

In cross-sectional analysis, participants with CPI4 had greater incidence risk for DMFS than participants with CPI<4 in both GLIDE and KNHANES. Participants with CPI4 had greater incidence risk for DFS than participants with CPI<4 in GLIDE, but reduced incidence risk for DFS than participants with CPI<4 in KNHANES (Table A2). These findings are consistent with the results of the main analysis, showing that measures of caries exposure are non-independent of periodontal status, with population-specific associations.

**TABLE A1.** Unadjusted and fully adjusted hazard ratios for CPI4 as a predictor of tooth loss, taken from longitudinal analysis in Swedish GLIDE. The fully adjusted hazard ratio is obtained from a multivariable model including adjustment for age, sex, smoking status, educational level, baseline DFS and baseline number of teeth.

| Exposure | Age Group | Reference | Unadjusted hazard Ratio (95% CI) | Unadjusted  P value | Fully adjusted hazard ratio (95% Ci) | Fully adjusted  P value |
| --- | --- | --- | --- | --- | --- | --- |
| CPI 4 | Under 45 | CPI < 4 | 2.07 (1.85, 2.32) | <0.0001 | 1.69 (1.50, 1.90) | <0.0001 |
|  | 45 to 54.9 |  | 1.76 (1.64, 1.89) | <0.0001 | 1.35 (1.25, 1.46) | <0.0001 |
|  | 55 or older |  | 1.50 (1.41, 1.60) | <0.0001 | 1.28 (1.21, 1.37) | <0.0001 |
|  | Combined |  | 1.66 (1.59, 1.73) | <0.0001 | 1.36 (1.30, 1.42) | <0.0001 |

| **GLIDE Cross-sectional analysis - Cases defined as CPI 4 in one or more sextants or 6mm pocketing around one or more teeth** | | | | | | | | |
| --- | --- | --- | --- | --- | --- | --- | --- | --- |
| **Age group** | **% of cases** | **Number** | **Fully adjusted ratio of index in cases:controls (95% CI)** | | | | | |
|  |  |  | **DMFS** | **DMFT** | **DFS** | **Standardized DFS** | **Number of surfaces** | **Number of teeth** |
| Under 45 | 12.67 | 10,073 | 1.05(1.01,1.09) | 1.03(1.00,1.05) | 1.01(0.97,1.05) | 1.02(0.99,1.06) | 0.99(0.97,1.00) | 0.99(0.99,1.00) |
| 45 to 54.9 | 25.55 | 9,794 | 1.09(1.07,1.12) | 1.04(1.03,1.06) | 1.03(1.00,1.05) | 1.06(1.03,1.08) | 0.98(0.97,0.99) | 0.98(0.97,0.98) |
| 55 or older | 42.54 | 8,824 | 1.04(1.02,1.05) | 1.01(1.00,1.02) | 1.01(0.99,1.03) | 1.02(1.01,1.04) | 0.98(0.98,0.99) | 0.99(0.98,0.99) |
| Overall | 26.25 | 28,691 | 1.05(1.04,1.07) | 1.02(1.01,1.03) | 1.01(1.00,1.03) | 1.03(1.02,1.05) | 0.98(0.98,0.98) | 0.98(0.98,0.98) |
| **KNHANES Cross-sectional analysis – Cases defined as CPI 4** | | | | | | | | |
| **Age group** | **% of cases** | **Number** | **Fully adjusted ratio of index in cases:controls (95% CI)** | | | | | |
|  |  |  | **DMFS** | **DMFT** | **DFS** | **Standardized DFS** | **Number of surfaces** | **Number of teeth** |
| Under 45 | 2.3 | 14,486 | 1.12(1.00,1.26) | 1.00(0.91,1.11) | 0.85(0.76,0.95) | 0.86(0.77,0.97) | 0.97(0.96,0.99) | 0.97(0.96,0.99) |
| 45 to 54.9 | 10.2 | 6,500 | 1.21(1.12,1.30) | 1.14(1.07,1.21) | 0.93(0.84,1.04) | 0.95(0.86,1.05) | 0.96(0.94,0.97) | 0.96(0.95,0.97) |
| 55 or older | 12.1 | 12,845 | 1.01(0.97,1.05) | 1.00(0.96,1.04) | 0.96(0.89,1.02) | 0.96(0.90,1.03) | 0.99(0.970,1.01) | 0.99(0.97,1.01) |
| Overall | 6.6 | 33,831 | 1.06(1.02,1.09) | 1.01(0.97,1.04) | 0.91(0.87,0.97) | 0.92(0.87,0.97) | 0.99(0.98,1.00) | 0.99(0.98,1.00) |

**TABLE A2.** Association between periodontal status and WHO caries indices in cross-sectional analysis. The estimates are incidence risk ratios obtained from multivariable Poisson regressions models which incorporate adjustment for age, sex, smoking status and educational level.

**References**

1. Hallmans, G. *et al.* Cardiovascular disease and diabetes in the Northern Sweden Health and Disease Study Cohort - evaluation of risk factors and their interactions. *Scandinavian journal of public health. Supplement* **61**, 18-24 (2003).

2. WHO. Oral health surveys: basic methods - 5th edition. (World Health Organization, <http://www.who.int/oral_health/publications/9789241548649/en/>, 2013).

3. Sejong, C. National Health Statistics 2013: Korean National Health and Nutritional Examination Survey (KNHANES VI-I). (Ministry of Health and Welfare, Korea Centers for Disease Control and Prevention, 2014).

4. Sejong, C. Standardization for oral health survey in KNHANES (2010). (Korea Centers for Disease Control and Prevention, 2011).
